# Geographic Variation in Chin Shape Challenges the Universal Facial Attractiveness Hypothesis

**DOI:** 10.1371/journal.pone.0060681

**Published:** 2013-04-03

**Authors:** Zaneta M. Thayer, Seth D. Dobson

**Affiliations:** 1 Department of Anthropology, Northwestern University, Evanston, Illinois, United States of America; 2 Department of Anthropology, Dartmouth College, Hanover, New Hampshire, United States of America; University of Pennsylvania, United States of America

## Abstract

The universal facial attractiveness (UFA) hypothesis proposes that some facial features are universally preferred because they are reliable signals of mate quality. The primary evidence for this hypothesis comes from cross-cultural studies of perceived attractiveness. However, these studies do not directly address patterns of morphological variation at the population level. An unanswered question is therefore: Are universally preferred facial phenotypes geographically invariant, as the UFA hypothesis implies? The purpose of our study is to evaluate this often overlooked aspect of the UFA hypothesis by examining patterns of geographic variation in chin shape. We collected symphyseal outlines from 180 recent human mandibles (90 male, 90 female) representing nine geographic regions. Elliptical Fourier functions analysis was used to quantify chin shape, and principle components analysis was used to compute shape descriptors. In contrast to the expectations of the UFA hypothesis, we found significant geographic differences in male and female chin shape. These findings are consistent with region-specific sexual selection and/or random genetic drift, but not universal sexual selection. We recommend that future studies of facial attractiveness take into consideration patterns of morphological variation within and between diverse human populations.

## Introduction

According to Darwin [1], [2], sexual selection results in the proliferation of phenotypes that provide a competitive advantage in the struggle to find mates. The two main modes of sexual selection are intrasexual competition, where individuals of the same sex compete for access to potential mates, and intersexual choice, where individuals vary in their ability to attract potential mates [3]. Most studies of sexual selection in humans have focused on mate choice in relation to physical attractiveness [4]. For example, it has been suggested that some facial phenotypes are universally preferred by the opposite sex because they are reliable indicators of mate quality [5]–[12]. Psychologists have attempted to test this universal facial attractiveness (UFA) hypothesis by assessing the degree of cross-cultural variation in facial preferences. These studies have shown that people from culturally diverse backgrounds tend to regard similar facial phenotypes as attractive [13]–[15], regardless of prior exposure to Western concepts of beauty [6], [16], [17]. However, a uniform pattern of preferences is only one prediction of the UFA hypothesis. If facial phenotypes are universally preferred and strongly selected, then patterns of morphological variation in those traits should be similar across geographic populations. We test this prediction using quantitative data on one of the phenotypes commonly discussed among proponents of the UFA hypothesis: the chin.

Chin shape is generally regarded as an important aspect of facial attractiveness, especially in males [18]. Some evidence suggests that males with broad chins are viewed as socially dominant across cultures [19], [20]. Broad-chinned males earn higher ranks in the military [21], are regarded as more masculine and attractive [9], [22]–[26], and have greater reproductive success in some societies [27], [28] than males with narrow chins. A preference for a broad male chin might be selected for through a "handicap model" of sexual selection [29]. This hypothesis is based on the observation that the chin is formed through the deposition of excess cortical bone during development [30]. Since cortical bone growth is stimulated by testosterone, and testosterone is immunosuppressive in high concentrations, the ability to have a broad chin and still be healthy is hypothesized to demonstrate high mate quality. It is important to note that studies of female attractiveness consistently report that a small or narrow chin is associated with a more feminine appearance [23], [26], [31], [32]. This reinforces the notion that a broad chin is a signal of masculinity and suggests that selection might be occurring in opposite directions between the sexes.

The hypothesis that chin shape has undergone sexual selection in humans is supported by recent evidence that males and females differ with regard to chin shape [33]. In a previous study [34], we developed a morphometric technique for quantifying chin shape using elliptical Fourier functions analysis, or EFFA [35]. To test for sex differences in chin shape we compared male and female chin surface contours in a pooled sample of human skeletons (N = 180) from nine geographic regions. We found that males differed significantly from females in having larger and more projecting lateral tubercles at the base of the chin [34]. These bony protuberances contribute to the “broad chin” phenotype that distinguishes males from females [36], [37]. Thus, we could not refute the UFA hypothesis because our results were consistent with the observation that females tend to prefer males with broad chins [14]. However, we observed a high degree of individual variation in chin shape in our sample, and some of this variation appeared to be geographically patterned.

Here we focus on the question of geographic variation in chin shape with respect to an evolutionary aspect of the UFA hypothesis that is often overlooked. If preferences for particular chin shapes are universal in the strict sense, *and these preferences influence the evolution of the chin*, then chin shapes should not differ significantly between geographic regions. Our results suggest that chin shape is geographically variable in both sexes, challenging the notion of universal sexual selection on chin shape.

## Materials and Methods

### Ethics statement

ZMT was granted permission to conduct an on-site analysis of mandibular specimens at the American Museum of Natural History (New York, NY).

### Skeletal sample

Specimens were randomly selected for study. We focused on nine Old-World geographic regions: Australia, eastern Africa, southern Africa, southeast Asia, central Asia, eastern Asia, northern Europe, eastern Europe and western Europe. Ten adult males and ten adult females were selected from each of these regions (N = 180). Pathological specimens, particularly those with alveolar resorption, were excluded from the study.

Because our study concerns inter-population variation in chin shape, we focused on sampling a wide range of geographic regions. As a consequence, our within-region, sex-specific sample sizes were relatively small (N = 10). Error variance due to small sample size increases the chances of a Type II Error, i.e., failing to reject a false null hypothesis. Thus, our sampling strategy had the effect of making our inter-population analyses more conservative.

### Quantifying chin shape

A detailed description of our approach to quantifying chin shape using elliptical Fourier functions analysis (EFFA) can be found in Thayer and Dobson [34]. In short, three parallel tracings of chin surface contours were taken from the right side of each mandibular symphysis. The first chin contour (midline contour, or MC) was taken at the midline of the symphysis, beginning on the anterior aspect of the digastric fossa and ending at infradentale. The second contour (canine contour, or CC) was taken at the medial aspect of the canine, parallel to the midline. The last contour (incisor contour, or IC) was taken halfway between the first two tracings, as determined by digital calipers.

Each chin contour tracing was scanned, mirror imaged to form a closed ellipse, and then digitized to perform the EFFA. Fourier analysis is a technique for computing a trigonometric function that can be used to describe the shape of curve, or in this case an ellipse. Curvature is quantified through the use of sine and cosine terms that describe the repeated elements (i.e., harmonics) in a sinusoidal waveform [38]. The actual values that are analyzed are the two amplitudes of each harmonic. These Fourier coefficients can be used as general measures of shape in subsequent multivariate statistical analyses [39]. EFFA produces a large number of harmonics for a given shape. We used only the first 20 harmonics, which resulted in 40 Fourier coefficients per harmonic, and a total of 800 coefficients per chin surface contour.

### Statistical analyses

Given the large number of Fourier shape descriptors per chin surface contour, we used principal components analysis (PCA) to further reduce the data. We performed PCAs separately for each contour (MC, CC, and IC) in each sex. Only components with eigenvalues greater than 1.0 were retained. This resulted in a total of 11 PCs for the female sample (3 MC, 4 CC, and 4 IC), and 10 PCs for the male sample (3 MC, 3 CC, and 4 IC) (Table S1). PC loadings were then used as variables in subsequent statistical tests.

We used multivariate analyses of variance (MANOVA) to test for significant geographic differences in chin shape among males and females. If MANOVA revealed a significant effect then pairwise comparisons between regions were made using Hotelling's t-squared statistic, which is a measure of the degree of multivariate difference between two groups. This was done to visually identify possible outlier regions for exclusion in subsequent reanalysis using MANOVA. No t-tests were performed in these post-hoc analyses. Thus, no corrections for multiple comparisons were necessary.

## Results

**Figure 1:**
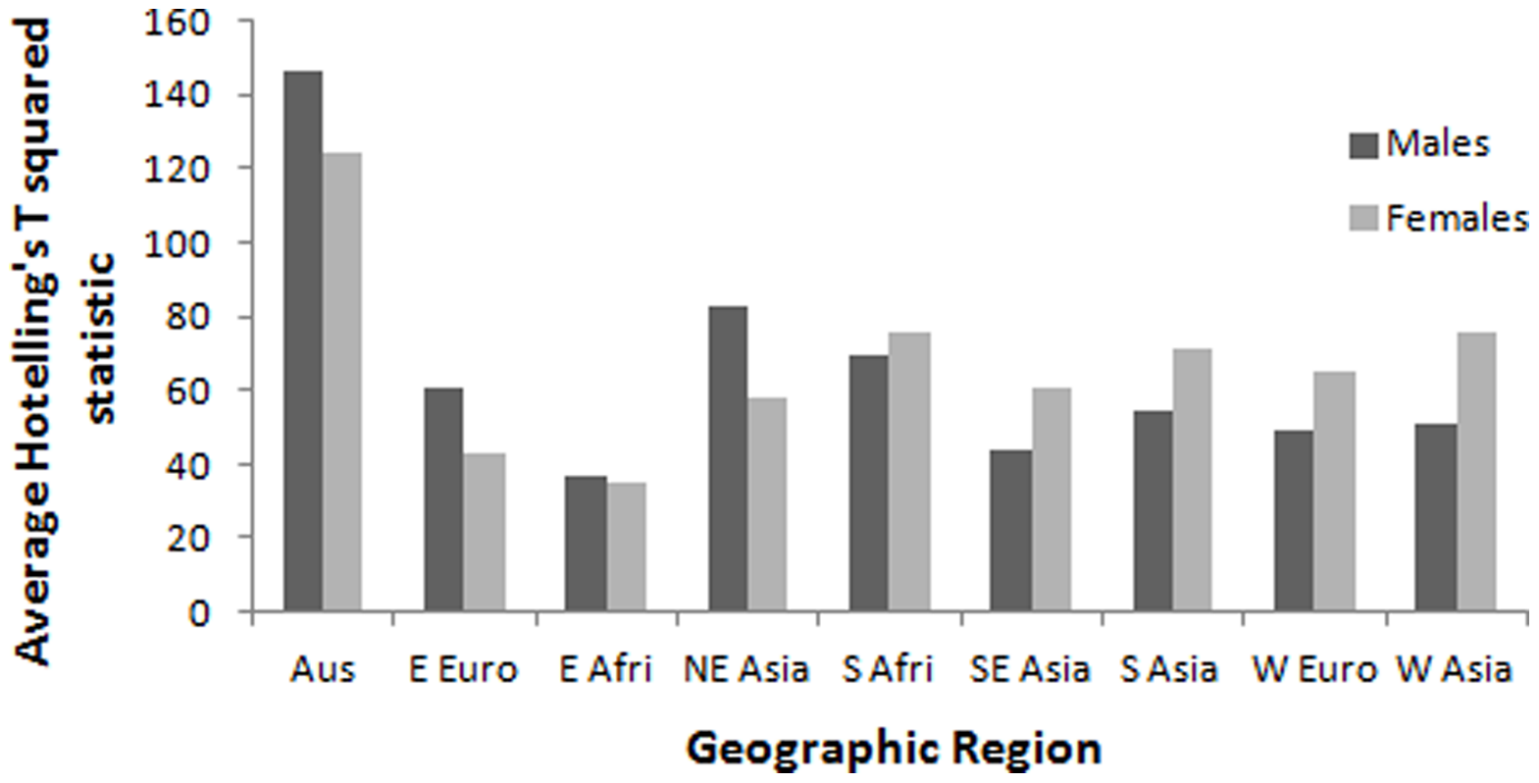
Hotelling's t-squared statistic reveals that the most pronounced pairwise multivariate differences in chin shape in our sample involve comparisons with Australia.

**Figure 2:**
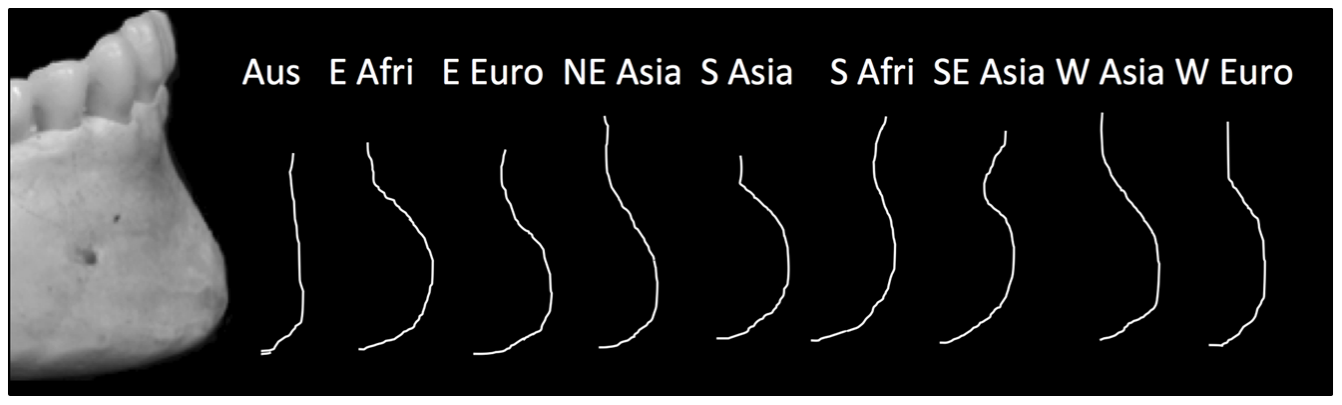
Examples of male incisor outlines from left to right: Australia (VL–245); Eastern Africa (VL – 4039); Eastern Europe (VL – 2327); Northeast Asia (99 – 8155); Southern Asia (VL – 3659); Southern Africa (99 – 8433); Southeast Asia (VL – 597); Western Asia (VL – 1268); Western Europe (VL – 3652).

MANOVA revealed a statistically significant effect of geographic region on male (Table 1) and female chin shape (Table 2). A graph of the average Hotelling's t-squared statistic revealed that the largest chin shape differences were observed in pairwise comparisons between Australia and all other regions (Figure 1). Visual inspection of the original chin surface contour tracings reinforced the status of Australia as a potential outlier (Figure 2). Both male and female Australians differed from other regions in having less projecting chins on average. Nevertheless, when we repeated the MANOVA after excluding the Australian sub-sample, the results were still statistically significant for both sexes (Table S2 & Table S3).

**Table 1:** Results of multivariate analysis of variance (MANOVA) in males with geographic region as a categorical predictor of chin shape.

Test statistic	Value	F-value	Hypothesized df	Error df	*P*
Pillai's Trace	1.53	1.87	8	81	0.000*
Wilks' Lambda	0.10	2.50	8	81	0.000*

* *P*<0.001

**Table 2:** Results of multivariate analysis of variance (MANOVA) in females with geographic region as a categorical predictor of chin shape.

Test statistic	Value	F-value	Hypothesized df	Error df	*P*
Pillai's Trace	1.43	1.55	8	81	0.0018*
Wilks' Lambda	0.16	1.72	8	81	0.0002*

* *P*<0.01

## Discussion

The results of our study suggest that chin shape is not universal in the strict sense because there were statistically significant differences between geographic regions in both sexes. This finding challenges an often-overlooked evolutionary prediction of the universal facial attractiveness (UFA) hypothesis, at least with regard to chin shape. If people from all over the world exhibit similar chin shape preferences, as the UFA hypothesis suggests [7], and if mating preferences influence morphological evolution, as implied by sexual selection theory, then we would expect to see negligible geographic variation in chin shape. Our results suggest that this is not the case.

However, chin shape is not entirely region specific either. If this were the true, then we would expect to see significant shape differences between all geographic pairings. Instead, we found that the most pronounced differences were observed in pairwise comparisons involving Australia (Figure 1). Nonetheless, when we excluded the Australian sub-sample from our analysis, we found statistically significant differences in chin shape among non-Australian populations as well. Thus, geographic variation in chin shape appears to be a general feature of recent human evolution. This observation, when combined with previous evidence of sexual dimorphism in chin shape [32], [33], suggests the possibility of region-specific chin shape preferences in some parts of the world.

For example, we hypothesize that native Australians have different facial preferences than other people, and that these differences are at least partially responsible for their “robust” craniofacial morphology [40]. Indeed, previous studies have demonstrated inter-population differences in human facial preferences [41]. However, to our knowledge, no systematic study of facial preferences in native Australians has ever been conducted. Given our results, one might predict that Australian women tend to prefer men with less prominent chins. The relatively high frequency of less prominent chins in Australia might also be due in part to an evolutionary history of genetic exchange between archaic and early modern human populations in Australasia during the Pleistocene (e.g., [42]). Archaic humans, such as *Homo erectus*, tended to have less prominent chins [43]. Thus, given that sexual selection is often frequency dependent [44], a genetic legacy from archaic *Homo* in Australasia might have biased female preferences toward less prominent chins in males. These hypotheses are in need of further examination.

Other adaptive explanations for chin shape variation have also been suggested, most notably the masticatory stress hypothesis [45]. According to this hypothesis, the chin functions as a buttress against mechanical stress caused by chewing. However, recent research suggests that this hypothesis is no longer viable since stress resistance is uncorrelated with chin shape in fossil and recent humans [43], [46]–[48]. Furthermore, the masticatory stress hypothesis does not explain the presence of sexual dimorphism in chin shape [33]. If chin shape differences were caused by differences in mechanical stress associated with different diets, then why would males and females differ significantly in chin shape within geographic regions? To our knowledge, there is no evidence suggesting that males and females from the same population eat foods that differ in their mechanical properties. Thus, we consider the masticatory stress hypothesis to be an unlikely alternative to region-specific sexual selection as an explanation for geographic variation in chin shape.

A more viable alternative explanation for geographic differences in chin shape is neutral rather than adaptive evolution. Some studies suggest that physical attractiveness is not necessarily a good predictor of mating patterns [44], [49]. This might be especially true of humans since mate choice is influenced by cultural factors as well as psychological predispositions in our species [50], [51]. Yu and Shepard [52] suggest that, at least in traditional societies, physical attractiveness might be less important as a determinant of mating patterns than cultural factors such as kinship. People in traditional societies also benefit from increased knowledge of potential mates due to more direct and prolonged social contact. Such familiarity might circumvent the need for facial signals of mate quality in these societies. Thus, chin shape differences might have evolved primarily as a result of genetic drift rather than selection. This hypothesis is supported by previous studies that demonstrate an important role for genetic drift in the evolution of human cranial diversity [53].

In summary, the results of our study suggest that chin shape is geographically variable in both males and females. This pattern could be explained by either region-specific mating preferences or random genetic drift. Either way, the lack of universality calls into question an important evolutionary aspect of the UFA hypothesis, i.e., that sexual selection influences phenotypic evolution. It might be the case that facial preferences are truly universal, as previous research suggests, but that these preferences are not strong enough to impact morphological evolution, at least for our skeletal measures of chin shape. Moreover, recent globalization of Western ideas of beauty and other contemporary cultural factors might be influencing chin shape preferences in ways that are not represented in our historical skeletal sample. Nevertheless, our recommendation is that future studies of facial preferences take into consideration the implied morphological impact of universal facial attractiveness.

## Supporting Information

Table S1 Percent variance explained by each retained principal component (PC). Significance values represent the results from an ANOVA analysis.
(DOC)

Table S2 Results of multivariate analysis of variance (MANOVA) in males with geographic region as a categorical predictor of chin shape after excluding the Australian sub-sample.
(DOC)

Table S3 Results of multivariate analysis of variance (MANOVA) in females with geographic region as a categorical predictor of chin shape after excluding the Australian sub-sample.
(DOC)
