# Supplementary material for: Geographic Variation in Chin Shape Challenges the Universal Facial Attractiveness Hypothesis
Source: PLoS One. 2013 Apr 3;8(4):e60681. doi: 10.1371/journal.pone.0060681 (PMC3616164; doi:10.1371/journal.pone.0060681)
Supplement: Table S1 — Percent variance explained by each retained principal component (PC). Significance values represent the results from an ANOVA analysis. (DOC) [file pone.0060681.s001.doc]

| **Sex** | *50%* | *Canine* | *Incisor* |
| --- | --- | --- | --- |
| ***Female*** | PC1: 50.4%*  PC2: 28.5%*  PC3: 11.8%* | PC1: 43.5%  PC2: 30.9%**  PC3: 13.9%  PC4: 5.7%** | PC1: 47.0%*  PC2: 28.9%**  PC3: 13.3%  PC4: 5.0% |
| ***Male*** | PC1: 53.9%**  PC2: 25.8%*  PC3: 10.4%** | PC1: 45.1%  PC2: 29.3%**  PC3: 13.0%*  PC4: 5.1% | PC1: 53.0%*  PC2: 27.2%**  PC3: 11.9%** |

* = p < 0.05

** = p < 0.005
