# Supplementary material for: Geographic Variation in Chin Shape Challenges the Universal Facial Attractiveness Hypothesis
Source: PLoS One. 2013 Apr 3;8(4):e60681. doi: 10.1371/journal.pone.0060681 (PMC3616164; doi:10.1371/journal.pone.0060681)
Supplement: Table S2 — Results of multivariate analysis of variance (MANOVA) in males with geographic region as a categorical predictor of chin shape after excluding the Australian sub-sample. (DOC) [file pone.0060681.s002.doc]

| Test statistic | Value | F-value | Hypothesized df | | Error df | *P* |
| --- | --- | --- | --- | --- | --- | --- |
| Pillai's Trace | 1.21 | 1.44 | 7 | 72 | | 0.015* |
| Wilks' Lambda | 0.22 | 1.57 | 7 | 72 | | 0.005* |

**P* < 0.05
