# Supplementary material for: Geographic Variation in Chin Shape Challenges the Universal Facial Attractiveness Hypothesis
Source: PLoS One. 2013 Apr 3;8(4):e60681. doi: 10.1371/journal.pone.0060681 (PMC3616164; doi:10.1371/journal.pone.0060681)
Supplement: Table S3 — Results of multivariate analysis of variance (MANOVA) in females with geographic region as a categorical predictor of chin shape after excluding the Australian sub-sample. (DOC) [file pone.0060681.s003.doc]

| Test statistic | Value | F-value | Hypothesized df | | Error df | *P* |
| --- | --- | --- | --- | --- | --- | --- |
| Pillai's Trace | 1.25 | 1.35 | 7 | 72 | | 0.035* |
| Wilks' Lambda | 0.22 | 1.41 | 7 | 72 | | 0.020* |

**P* < 0.05
